# Single‐Unit Activity in the Human Medial Temporal Lobe Reflects Reduced Error Monitoring During Mind Wandering

**DOI:** 10.1002/hipo.70125

**Published:** 2026-08-27

**Authors:** Thomas P. Reber, Leila Chaieb, Marlene Derner, Valeri Borger, Rainer Surges, Florian Mormann, Juergen Fell

**Affiliations:** ^1^ Department of Epileptology University Hospital Bonn Bonn Germany; ^2^ Faculty of Psychology UniDistance Suisse Brig Switzerland; ^3^ Department of Neurosurgery University Hospital Bonn Bonn Germany

**Keywords:** experience sampling, hippocampus, medial temporal lobe, mind wandering, single‐unit recordings, spontaneous thought, sustained attention‐to‐response task

## Abstract

Mind wandering (MW) has been frequently reported to impair task performance. In this study, we investigated the hypothesis that it also affects error monitoring and adjustment. Several recent studies suggest that the hippocampus and adjacent regions significantly contribute to error monitoring. Therefore, we analyzed single‐unit recordings from the medial temporal lobe (MTL) in presurgical epilepsy patients performing an attention task, which included interspersed experience sampling probes. Behavioral performance data revealed reduced error adjustment during periods of MW compared to task‐focused attention. Analysis of single‐unit recordings showed population responses related to performance errors within the hippocampus and adjacent regions, supporting the idea that MTL regions contribute to error monitoring. Importantly, this error‐related population activity was significantly reduced during periods of MW compared to task focus. Together, our findings clearly support the hypothesis that MW impairs both error monitoring and adjustment.

## 1 Introduction

Feedback from errors is crucial for behavioral adjustment and learning (e.g., Metcalfe 2017). The brain generates intrinsic signals in response to erroneous task performance, most prominently, the error‐related negativity (Falkenstein et al. 1991; Gehring et al. 1993), as well as error‐related responses in different EEG frequency bands (e.g., Yordanova et al. 2004). The error‐monitoring network is thought to primarily involve the anterior cingulate cortex (ACC), prefrontal areas, anterior insula, and parietal lobe (e.g., Ullsperger et al. 2014). However, recent studies indicate that this network extends to MTL areas. Error‐related activity in the hippocampus and adjacent structures has been observed in humans through functional magnetic resonance imaging (Hargreaves et al. 2012) and intracranial EEG recordings (Völker et al. 2018), as well as in monkeys through local field potential recordings (Hargreaves et al. 2012). Importantly, single‐unit recordings in monkeys have revealed error‐related responses at both the unit and population level in the entorhinal cortex (Ku et al. 2021) and hippocampus (Ku et al. 2021; Wirth et al. 2009).

Mind wandering (MW) can be broadly defined as a shift of attention away from the current task or situation (Schooler et al. 2011). It primarily involves executive control and default mode areas, including the hippocampus and neighboring regions (Fox et al. 2015). MW has been linked to performance deficits in attentional and perceptual tasks (e.g., Leszczynski et al. 2017; Seli et al. 2013), as well as an increased likelihood of errors at work and accidents (e.g., Gil‐Jardiné et al. 2017; Yaor and Toker 2020). However, its effect on the internal monitoring of errors remains unclear. In a pioneering surface EEG study, Kam et al. (2012) investigated error monitoring and adjustment in a time‐estimation task requiring subjects to press a button exactly 1 s after an auditory cue. They observed that the fronto‐central error‐related negativity was attenuated, and error‐related motor adjustments were reduced during MW compared to on‐task focus.

In the present study, we examined the impact of MW on error monitoring and adjustment in a sustained attention‐to‐response task (SART) based on human single‐neuron recordings from the MTL. Specifically, we investigated whether there is behavioral evidence for impaired error adjustment in terms of post‐error accuracy and post‐error slowing during MW, and whether error‐related population activity in the hippocampus and adjacent regions is affected by MW.

### 1.1 Behavioral Data

Figure 2 shows the mean accuracies for non‐target and target trials (Figure 2A), as well as average reaction times for correct non‐target and incorrect target responses (in both cases, responses were button presses; Figure 2B). There was no significant difference in accuracy between non‐target and target trials (mean ± s.e.m.: 0.91 ± 0.02 vs. 0.90 ± 0.01; paired two‐tailed *t*‐test, *p* = 0.615). The mean reaction time was significantly longer for correct responses to non‐targets than for incorrect responses to targets (mean ± s.e.m.: 503 ± 20 ms vs. 378 ± 11 ms; *p* = 0.0006). Average MW rate across patients was 32.9% (±7.9% s.e.m). There was no significant difference in reaction times for correct non‐target responses between MW and on‐task periods (mean ± s.e.m.: 471 ± 22 ms vs. 467 ± 21 ms; *p* = 0.67).

### 1.2 Behavioral Analysis of Error Adjustment

**FIGURE 1:**
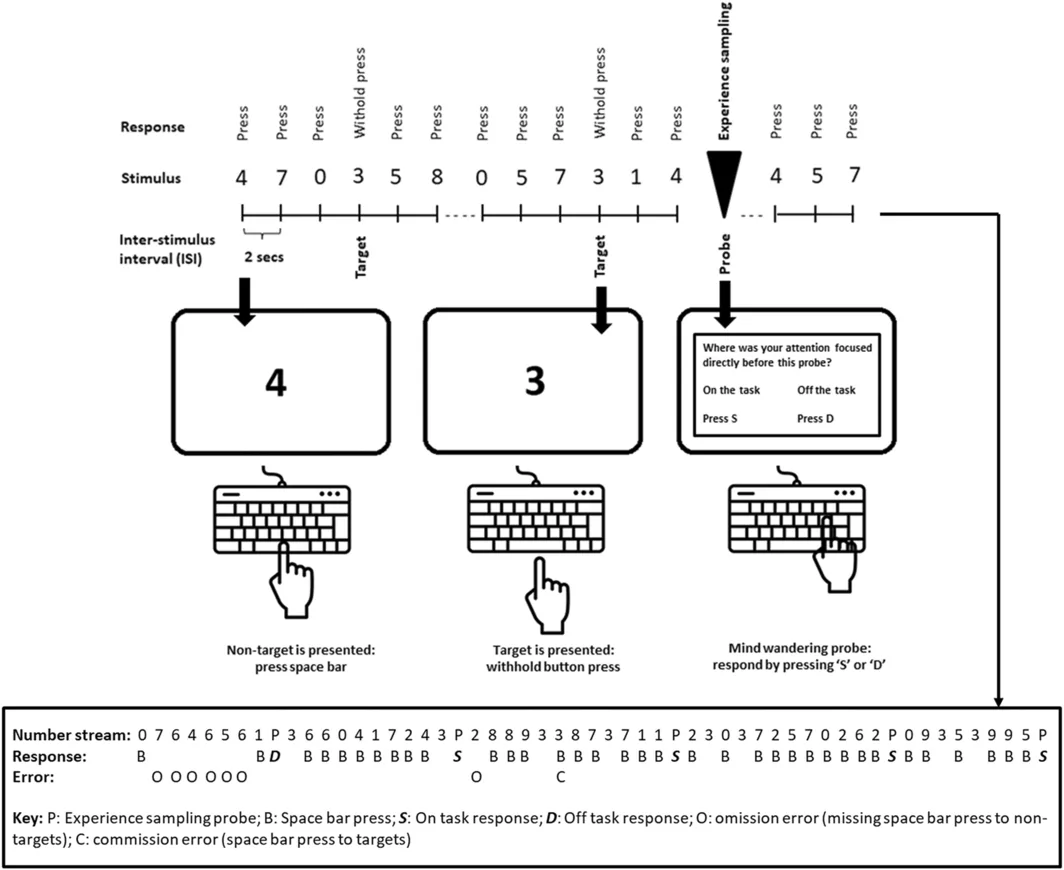
Schematic of the experimental paradigm (SART) with embedded experience sampling probes including screen shots (middle) and instructions (below). The panel beneath the main figure shows the first 50 trials of the SART recorded from a patient. The behavioral data shown here were obtained from the same patient whose unit activity is depicted in Figures 3A,B.

**FIGURE 2:**
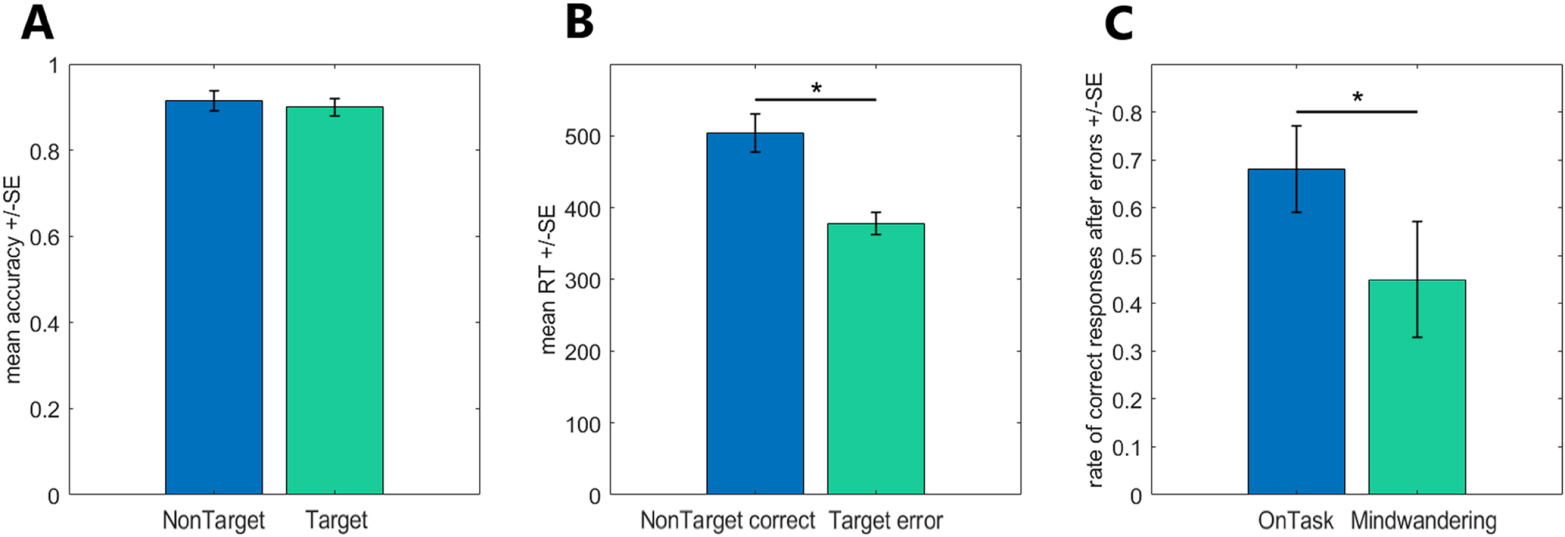
(A) Mean accuracy for non‐target and target trials (left), (B) mean reaction time (RT) for correct target trials and incorrect non‐target trials (middle), and (C) rate of correct responses after errors for on‐task periods and mind wandering (right). Asterisks indicate significance.

This analysis aimed to test the hypothesis that behavioral adjustment is poorer during MW compared to on‐task periods, as indicated by lower rates of correct responses to non‐targets immediately (i.e., on the first subsequent trial) after error responses to non‐targets (see Figure 1 for experimental paradigm). In support of this hypothesis, rates of correct responses to non‐targets immediately after non‐target errors were significantly lower during MW compared to on‐task periods (mean ± s.e.m.: 0.45 ± 0.12 vs. 0.68 ± 0.09; paired one‐tailed *t*‐test, *p* = 0.030; see Figure 2C). Rates of correct responses immediately following correct ones were not significantly lower during MW versus on‐task periods (mean ± s.e.m.: 0.96 ± 0.01 vs. 0.97 ± 0.02; paired one‐tailed *t*‐test, *p* = 0.243). There was a trend for higher non‐target error rates during MW compared to on‐task periods (mean ± s.e.m.: 0.16 ± 0.07 vs. 0.06 ± 0.03; paired one‐tailed *t*‐test, *p* = 0.063).

To examine how sensitive these results are to the size of the inclusion window (12 s), we additionally calculated the rates of correct responses immediately after errors for alternative inclusion windows of 16, 14, 10, and 8 s. Mean rate ± s.e.m. of correct responses immediately after errors during MW versus on‐task periods for the 16‐s window were: 0.49 ± 0.11 vs. 0.65 ± 0.09 (*p* = 0.087), for the 14‐s window: 0.45 ± 0.12 vs. 0.60 ± 0.09 (*p* = 0.109), for the 10‐s window: 0.53 ± 0.14 vs. 0.68 ± 0.10 (*p* = 0.080), and for the 8‐s window: 0.41 ± 0.13 vs. 0.62 ± 0.10 (*p* = 0.075).

### 1.3 Behavioral Analysis of Error Clusters

Based on the identification of non‐target error clusters, defined as sequences of non‐target errors immediately following each other, we observed a trend toward a larger average cluster size (including clusters with a size ≥ 1) during MW compared to on‐task periods (mean ± s.e.m.: 2.45 ± 0.56 vs. 1.46 ± 0.21; paired one‐tailed *t*‐test, *p* = 0.061).

To examine how sensitive these results are to the size of the inclusion window, we additionally calculated the average cluster size for inclusion windows of 16, 14, 10, and 8 s. Mean rates ± s.e.m. of the average cluster size during MW versus on‐task periods for the 16‐s window were: 2.53 ± 0.66 vs. 1.53 ± 0.25 (*p* = 0.075), for the 14‐s window: 2.70 ± 0.68 vs. 1.59 ± 0.25 (*p* = 0.077), for the 10‐s window: 2.10 ± 0.45 vs. 1.46 ± 0.20 (*p* = 0.095), and for the 8‐s window: 1.92 ± 0.35 vs. 1.42 ± 0.18 (*p* = 0.109).

Moreover, we found a significantly higher proportion of errors occurring within clusters of size ≥ 2 relative to the total number of errors during MW versus on‐task periods (mean ± s.e.m.: 0.59 ± 0.12 vs. 0.38 ± 0.11; *p* = 0.042). These results are consistent with the observation of reduced rates of correct responses after errors during MW, compared to on‐task periods.

To examine how sensitive these results are to the size of the inclusion window, we additionally calculated the proportion of errors within clusters of size ≥ 2 for inclusion windows of 16, 14, 10, and 8 s. Mean rates ± s.e.m. of the proportion of errors during MW versus on‐task periods for the 16‐s window were: 0.54 ± 0.11 vs. 0.40 ± 0.10 (*p* = 0.118), for the 14‐s window: 0.59 ± 0.12 vs. 0.46 ± 0.10 (*p* = 0.160), for the 10‐s window: 0.47 ± 0.14 vs. 0.37 ± 0.12 (*p* = 0.194), and for the 8‐s window: 0.51 ± 0.14 vs. 0.40 ± 0.12 (*p* = 0.204).

### 1.4 Behavioral Analysis of Post‐Error Slowing

This analysis evaluated reaction time differences between non‐target trials immediately following and preceding non‐target error trials. There was no significant difference in reaction time changes between MW and on‐task periods (mean ± s.e.m.: −37.1 ± 56.3 vs. −5.0 ± 74.7 ms; paired one‐tailed *t*‐test, *p* = 0.230).

To examine how sensitive these results are to the size of the inclusion window, we additionally calculated post‐error slowing for inclusion windows of 16, 14, 10, and 8 s. Reaction time changes (mean ± s.e.m.) during MW versus on‐task periods were not significant for the 16‐s window: −14.5 ± 43.3 vs. −6.6 ± 73.7 ms (*p* = 0.448), and for the 14‐s window: −22.8 ± 58.0 vs. −5.0 ± 73.9 ms (*p* = 0.379). However, for the 10‐s window: −66.0 ± 55.9 vs. 75.6 ± 64.0 (*p* = 0.015), and for the 8‐s window: −11.8 ± 11.4 vs. 135.7 ± 62.1 (*p* = 0.043), we observed evidence for diminished post‐error slowing during MW compared to on‐task periods, in line with the hypothesis of reduced error adjustment during MW.

### 1.5 Identification of Single Units

**FIGURE 3:**
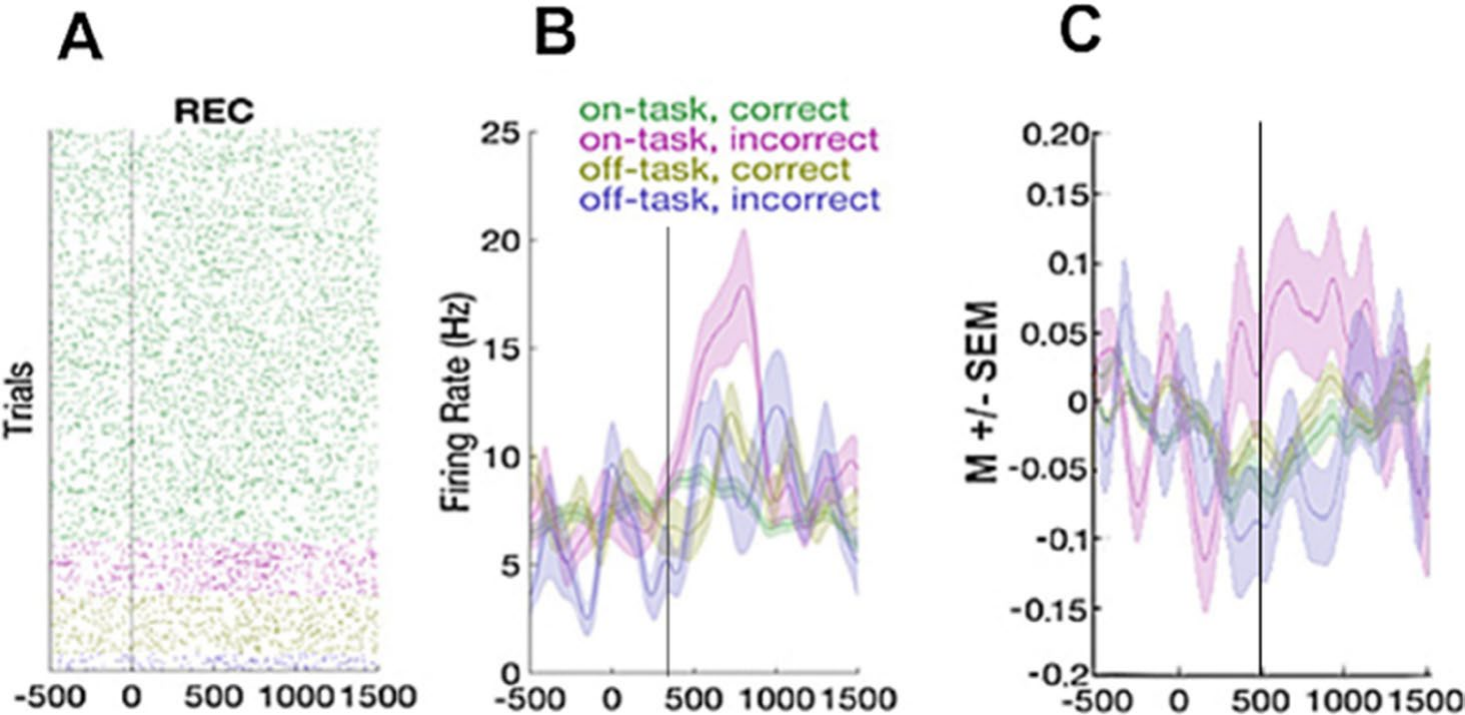
Time course of single unit and population activity (stimulus onset: time point 0): (A) raster plot (left), and (B) instantaneous firing rate (middle) of a unit in the right entorhinal cortex that increases its firing rate following incorrect non‐target responses (i.e., missed button presses) during on‐task periods; (C) averages and s.e.m. of z‐scored population activity across all analyzed units (N = 1457; right). Black vertical lines in panels B and C indicate average reaction times.

Based on semi‐automatic spike detection and sorting and manual curation (see Detailed Methods below), 3190 units were initially identified. We then selected recordings in which at least one non‐target trial was obtained in each condition of interest, i.e., on‐task correct, on‐task incorrect, off‐task correct and off‐task incorrect. The included 1457 units were recorded in 9 patients and were located in the amygdala (349 units, 24%), hippocampus (434 units, 30%), piriform cortex (132 units, 9%), parahippocampal cortex (264, 18%), and entorhinal cortex (278, 19%). The following analyses are based on firing rates during the time window [0.1 s; 1 s] after non‐target presentation. As an example, Figures 3A,B depict the raster plot and average correct and incorrect non‐target responses of a unit in the right entorhinal cortex during on‐task and off‐task (MW) periods. In addition, Figure 3C shows the average *z*‐scored population activity across all analyzed units.

### 1.6 Single‐Neuron Activity

**FIGURE 4:**
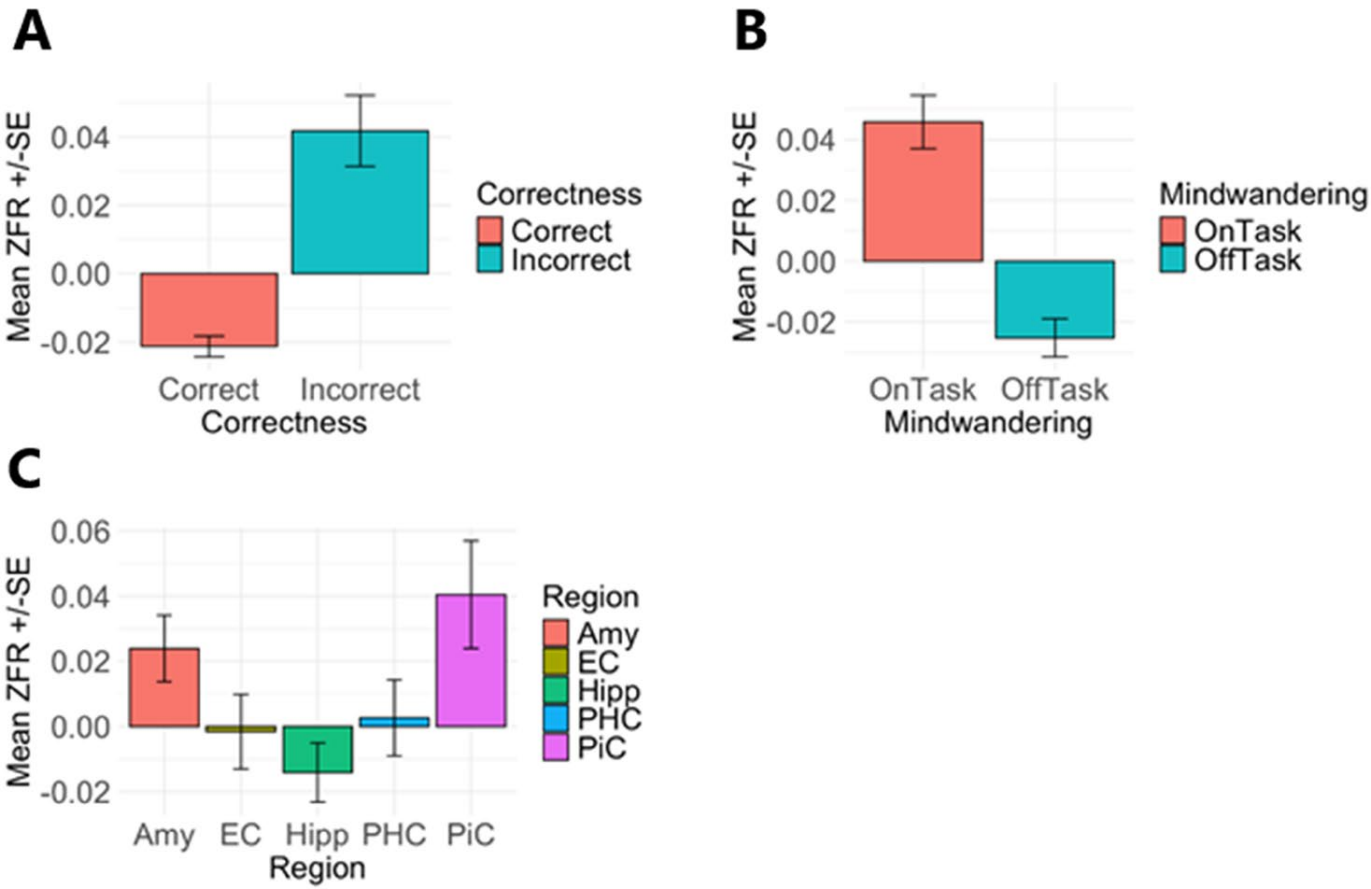
ANOVA results for population activity: statistically significant main effects for (A) CORRECTNESS (top left), (B) MIND WANDERING (top right), and (C) REGION (bottom left). Averages and s.e.m. of z‐scored population activity are depicted.

**FIGURE 5:**
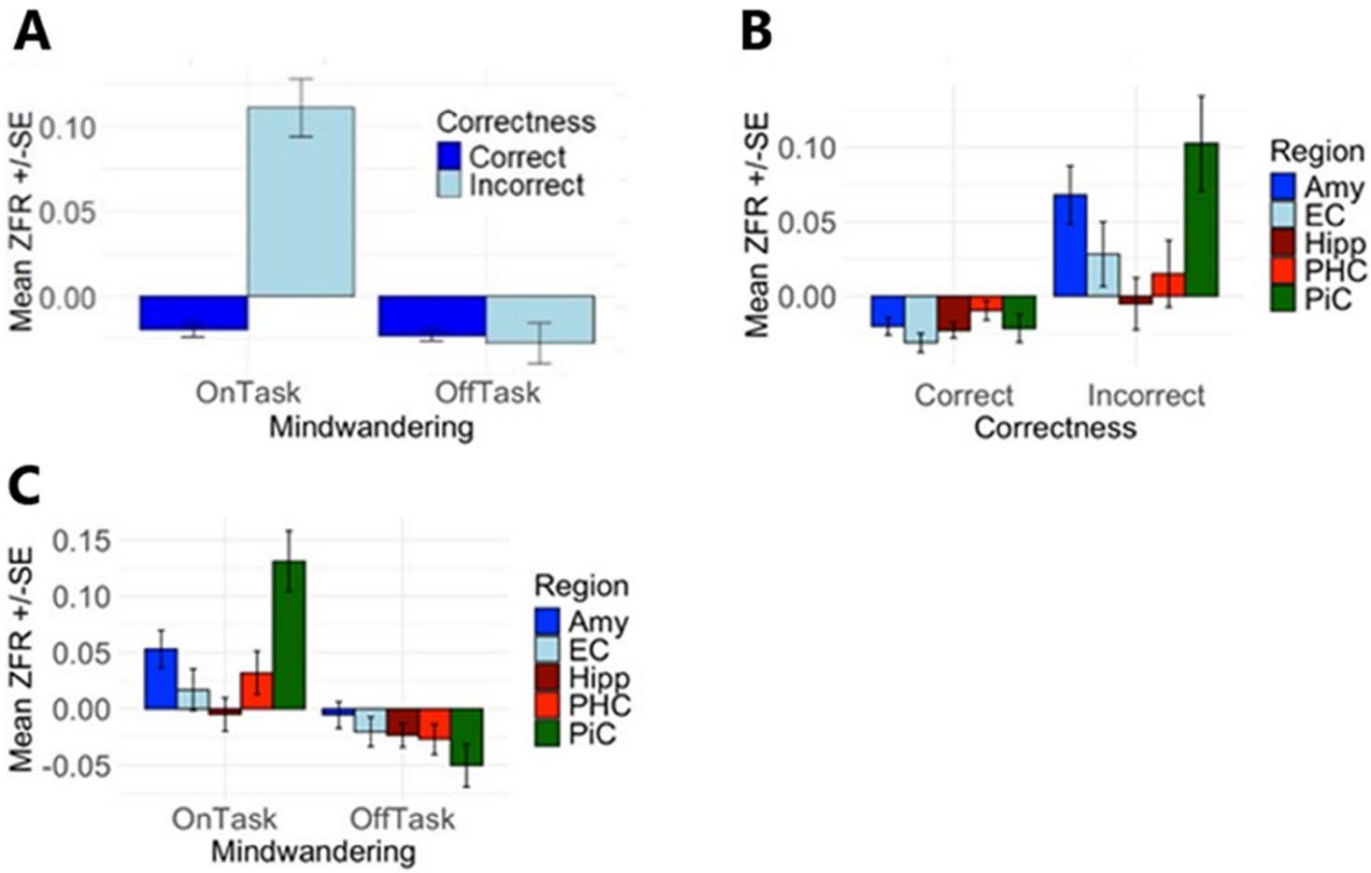
ANOVA results for population activity: statistically significant interactions between (A) CORRECTNESS and MIND WANDERING (top left), (B) CORRECTNESS and REGION (top right), as well as (C) MIND WANDERING and REGION (bottom left). Averages and s.e.m. of z‐scored population activity are depicted.

**FIGURE 6:**
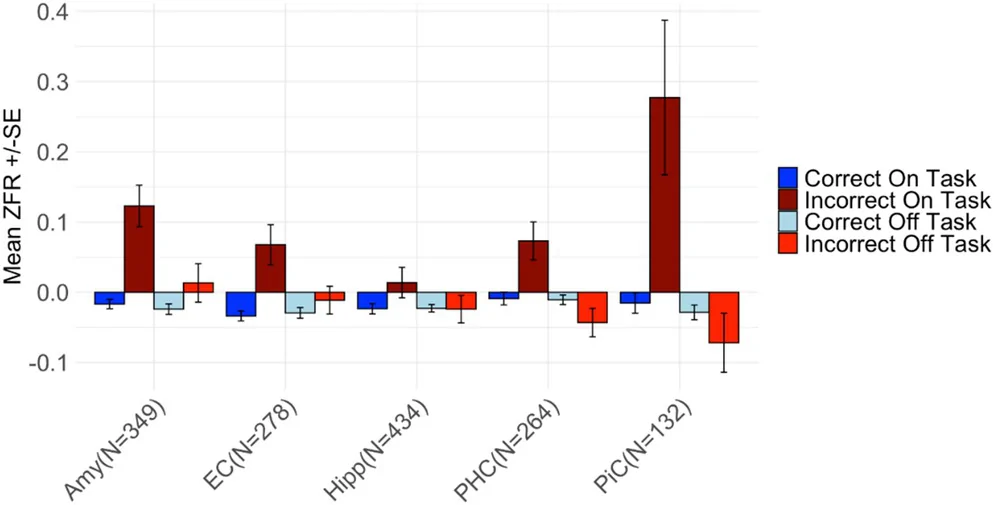
ANOVA results for population activity: Statistically significant three‐way interaction between correctness, mind wandering, and region. Averages and s.e.m. of z‐scored population activity are depicted.

To examine whether population responses at the single‐neuron level to the non‐targets were influenced by response correctness and MW, we conducted an ANOVA on firing rates, with CORRECTNESS (correct/incorrect) and MIND WANDERING (off‐task, on‐task) as repeated‐measures factors, and REGION as between‐units factor. This analysis revealed significant main effects for CORRECTNESS (*F*_1,1452_ = 33.66, *p* < 0.001), MIND WANDERING (*F*_1,1452_ = 43.54, *p* < 0.001), and REGION (*F*_4,1452_ = 3.21, *p* = 0.012; see Figure 4). Firing rates were higher for incorrect versus correct responses (Figure 4A), as well as for task focus compared to MW (Figure 4B). Across regions, the largest firing rates were observed within the piriform cortex and amygdala (Figure 4C). Importantly, the ANOVA indicated a highly significant interaction between CORRECTNESS AND MIND WANDERING (*F*_1,1452_ = 42.87, *p* < 0.001; see Figure 5A). During on‐task focus, firing rates were significantly larger for incorrect compared to correct responses (Tukey's HSD post hoc test, mean difference = 0.13, SE = 0.017, *t*_1452_ = 7.569, *p* < 0.001). However, firing rates for incorrect versus correct responses did not differ during MW (Tukey's HSD, mean difference = 0.004, SE = 0.012, *t*_1452_ = 0.355, *p* = 0.985). Additionally, there were significant interactions between CORRECTNESS and REGION (*F*_4,1452_ = 3.18, *p* = 0.013; see Figure 5B), and between MIND WANDERING and REGION (*F*_4,1452_ = 4.84, *p* < 0.001; see Figure 5C), as well as a significant three‐way interaction between CORRECTNESS, MIND WANDERING and REGION (*F*_4,1452_ = 4.33, *p* = 0.002; see Figure 6). Reflecting this three‐way interaction, firing rate differences between incorrect and correct responses were reduced during MW compared to task focus in the amygdala, entorhinal cortex and hippocampus, and were even inverted in the parahippocampal cortex and piriform cortex (Figure 5).

### 1.7 Error‐Related Target and Non‐Target Responses

**FIGURE 7:**
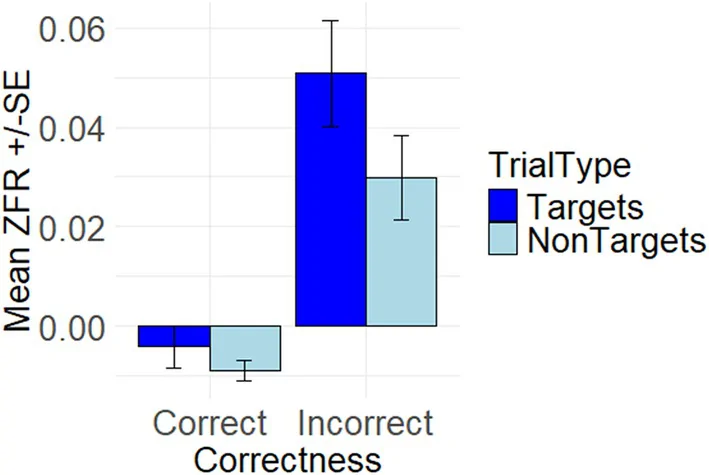
ANOVA results for population activity: Statistically significant main effect of correctness, and absent interaction with trial type. Averages and s.e.m. of *z*‐scored population activity are depicted.

The main analyses were restricted to non‐target trials as targets occurred rarely (i.e., at one‐tenth the rate of non‐targets). Including target trials would have resulted in an excessive number of missing data points when analyzing the correct versus incorrect and task focus versus MW contrasts within the 12‐s intervals preceding the experience sampling probes. A potential limitation of the chosen approach is that errors in non‐target trials are always associated with missing motor responses. Thus, neural correlates of motor action could, in principle, constitute a confound. To address this issue, we analyzed error‐related responses for both target trials (where errors are associated with motor responses) and non‐target trials (where errors are associated with the absence of motor responses). As this analysis was not restricted to specific time intervals (to reach sufficient trial numbers) and did not require a sufficient number of on‐task or off‐task responses, we could include data from 17 patients (2605 units). This analysis revealed similar error‐related responses for both target and non‐target trials (see Figure 7). A 2 × 2 repeated‐measures ANOVA on *z*‐scored firing rates, with the factors CORRECTNESS (correct, incorrect) and TRIAL TYPE (target, non‐target), showed a significant main effect of CORRECTNESS (*F*_1,2604_ = 45.77, *p* < 0.001). There was neither a main effect of TRIAL TYPE (*F*_1,2604_ = 0.45, *p* = 0.504), nor a significant interaction between CORRECTNESS and TRIAL TYPE (*F*_1,2604_ = 0.04, *p* = 0.833). These results support the interpretation that the observed error‐related effects are driven by response correctness rather than by motor action.

To conclude, we observed behavioral evidence for poorer error adjustment during MW compared to on‐task periods in a SART paradigm. Consistent with this behavioral finding, we also detected evidence of diminished error monitoring based on single‐unit population activity within the hippocampus and adjacent regions. Both results align with our hypotheses, and are in accordance with a previous surface EEG study (Kam et al. 2012) that reported decreased error monitoring and adjustment during MW versus on‐task focus in a time‐estimation task. Furthermore, our data clearly support the idea that medial temporal regions contribute to error monitoring (Hargreaves et al. 2012; Ku et al. 2021; Völker et al. 2018; Wirth et al. 2009). A likely primary source of the error signals is the posterior medial frontal cortex (pMFC; for overviews, see e.g., Ullsperger et al. 2014; Ridderinkhof et al. 2004). Based on single‐unit and intracranial EEG recordings in humans, it has for instance, been demonstrated that error monitoring is reflected in activity in the pre‐supplementary motor area and the dorsal anterior cingulate cortex, both subregions of the pMFC (Fu et al. 2019; Pourtois et al. 2010). However, intracranial EEG recordings have also suggested that the hippocampus and amygdala are part of a more extended network involved in error monitoring (Völker et al. 2018; Pourtois et al. 2010).

In summary, although it has been frequently reported that MW impairs task performance (e.g., Gil‐Jardiné et al. 2017; Leszczynski et al. 2017; Seli et al. 2013; Yaor and Toker 2020), our findings go beyond this notion and demonstrate that MW also significantly affects error monitoring and adjustment at the neuronal and behavioral levels.

## 2 Detailed Methods

### 2.1 Participants

Microwire recordings from 17 epilepsy patients (12 female; mean age: 49.7 ± 10.5 years) undergoing presurgical evaluation were analyzed. Mediotemporal depth electrodes and microwires had been implanted for chronic seizure monitoring and evaluation for epilepsy surgery. All patients gave informed written consent. The study conformed to the guidelines of the Medical Institutional Review Board at the University of Bonn (Ethics Number: 095/10 and 248/11).

### 2.2 Experimental Paradigm: Sustained Attention to Response Task

Patients performed a go/no‐go task—a variant of the SART (SART; Robertson et al. 1997). They responded to a continuous stream of digits presented onscreen and pressed the space bar whenever a non‐target number (0–2; 4–9) was shown and withheld the button press whenever a “3” appeared (target) (Figure 1). The assigned target number (3) never varied during the paradigm. Before the experiment began, patients were instructed to respond as quickly and as accurately as possible. No feedback was given to the patients after each trial as to correct or incorrect responses. The experimental paradigm proceeded automatically after each button press. Stimuli were presented until button press for a maximum of 1 s, with a 2 s inter‐stimulus interval. Each patient performed the task until they felt fatigued or unable to continue, or had reached 120 experience sampling probes (around 70 min), which was the conclusion of the paradigm (maximum 120 probes).

### 2.3 Experience Sampling

The patients' propensity to mind‐wander was examined using experience sampling probes intermittently embedded within the SART digit stream (Figure 1). The probe addressed the focus of the patient's attention immediately prior to the presentation of the probe. Inter‐probe intervals randomly varied between 25 to 35 s. When the probe appeared, the patients were prompted onscreen to answer the question “Where was your attention focused directly before this probe?” They could respond with either “On the task” (by pressing the S key) or “Off the task” (by pressing the D key). “On the task” responses indicated attentiveness to the SART, while “Off the task” responses were evaluated as episodes of self‐reported MW.

### 2.4 Data Recording

Recordings were obtained from a bundle of nine microwires (eight high‐impedance recording electrodes, one low‐impedance reference, AdTech, Racine, WI) protruding from the end of each depth electrode targeting hippocampus (11 patients), entorhinal cortex (10 patients), amygdala (10 patients), parahippocampal cortex (10 patients), and piriform cortex (4 patients). Within the hippocampus, the anterior and middle third were targeted. MTL recording microwires per patient ranged between 8 and 96 (referenced against one of the reference microwires). The differential signal from the microwires was amplified using a Neuralynx ATLAS system (Bozeman, MT), filtered between 0.1 and 9000 Hz, and sampled at 32 kHz. Signals were band‐pass filtered between 300 and 3000 Hz. Spike detection and sorting was performed using the Combinato software package (Niediek et al. 2016). After automated sorting using standard parameters, clusters in every channel were manually adjusted based on cluster shape, cross correlograms, and other features. Sorted units were classified as single units, multiunits, or artifacts based on spike shape and variance, ratio between spike peak value and noise level, the inter‐spike interval distribution, and presence of a refractory period for the single units (Mormann et al. 2011; Quiroga et al. 2004; Reber et al. 2017).

### 2.5 Labeling of Time Intervals as On‐Task Versus Off‐Task

On‐task (MW) and off‐task periods were identified based on responses to experience sampling probes. For both the behavioral evaluation of error adjustment and the analysis of single‐unit firing rates (see below), a 12‐s inclusion window preceding each probe was used. This window corresponded to six consecutive responses to number stimuli. The chosen window length is identical to that used by Kam et al. (2012) and corresponds to the assumed periodicity of dynamic shifts between MW and task‐focused attention (Sonuga‐Barke and Castellanos 2007).

### 2.6 Behavioral Analysis of Error Adjustment

This analysis was conducted on a larger sample of 23 patients (16 female; mean age: 47.4 ± 11.6 years) with intracranial recordings (of which only 17 had microwires) performing the SART. For the non‐target stimuli, we analyzed whether the rates of correct responses immediately following errors and immediately following correct responses, as well as the error rates, differed between MW and on‐task periods. To calculate response rates, the numbers of correct non‐target responses immediately following non‐target errors and correct non‐target responses were divided by the total number of non‐target responses immediately following non‐target errors/correct responses within the inclusion window. To calculate error rates, the number of non‐target errors was divided by the total number of non‐target responses. Response rates and error rates during MW and on‐task periods were compared using paired *t*‐tests. These *t*‐tests were based on patients with sufficient numbers of non‐target responses (i.e., null values were excluded): 9 patients for the rate of correct responses after errors, 15 patients for the rate of correct responses after correct responses, and 16 patients for the error rate.

### 2.7 Behavioral Analysis of Error Clusters

We identified the sizes of non‐target error clusters, defined as sequences of non‐target errors immediately following each other. For each patient, we then calculated two measures: (a) the average size of non‐target error clusters, including clusters with a size ≥ 1; and (b) the proportion of errors within clusters of size ≥ 2 relative to the total number of errors. *t*‐tests were based on patients with sufficient numbers of non‐target responses (*n* = 10).

### 2.8 Behavioral Analysis of Post‐Error Slowing

For non‐target trials with errors, the reaction time for the non‐target trial immediately preceding the error trial was subtracted from the reaction time for the non‐target trial immediately following it. This difference served as a measure of post‐error slowing (i.e., positive values indicate post‐error slowing). *t*‐tests were based on patients with a sufficient number of non‐target responses (*n* = 9).

### 2.9 Computation of Average Firing Rates

Data analysis was conducted in MATLAB. For the analysis of firing rates, non‐target trials requiring a button press response were selected. Spike counts were obtained for the interval from 100 to 1000 ms after stimulus presentation. Mean firing rates across trials were calculated separately for trials with correct (button press) and incorrect (no button press) responses during on‐task and off‐task (MW) periods.

### 2.10 Statistical Evaluation of Population Activity

Using R, mixed ANOVAs were conducted with the repeated‐measure factors CORRECTNESS (correct, incorrect) and MIND WANDERING (on‐task, off‐task), as well as the between‐units factor REGION (hippocampus, entorhinal cortex, amygdala, parahippocampal cortex, piriform cortex), to explore the influences of response correctness, MW, and brain region on single‐neuron responses. Only sessions containing at least one non‐target trial for each of the four combined conditions correct/on‐task, correct/off‐task, incorrect/on‐task, and incorrect/off‐task were included in this analysis, which resulted in the inclusion of 9 patients. For post hoc comparisons, two‐sided Tukey's Honestly Significant Difference (HSD) tests were calculated.

## Funding

This work was supported by Deutsche Forschungsgemeinschaft (DFG FE‐366/11‐1, MO 930/4‐2, SPP 2205, SFB 1089).

## Conflicts of Interest

The authors declare no conflicts of interest.

## Data Availability

The data that support the findings of this study are available from the corresponding author upon reasonable request.

## References

[hipo70125-bib-0001] Falkenstein, M. , J. Hohnsbein , J. Hoormann , and L. Blanke . 1991. “Effects of Crossmodal Divided Attention on Late ERP Components. II. Error Processing in Choice Reaction Tasks.” Electroencephalography and Clinical Neurophysiology 78, no. 6: 447–455. 1712280 10.1016/0013-4694(91)90062-9

[hipo70125-bib-0002] Fox, K. C. R. , R. N. Spreng , M. Ellamil , J. R. Andrews‐Hanna , and K. Christoff . 2015. “The Wandering Brain: Meta‐Analysis of Functional Neuroimaging Studies of Mind‐Wandering and Related Spontaneous Thought Processes.” NeuroImage 111: 611–621. 25725466 10.1016/j.neuroimage.2015.02.039

[hipo70125-bib-0003] Fu, Z. , D.‐A. J. Wu , I. Ross , et al. 2019. “Single‐Neuron Correlates of Error Monitoring and Post‐Error Adjustments in Human Medial Frontal Cortex.” Neuron 101, no. 1: 165–177. 30528064 10.1016/j.neuron.2018.11.016 PMC6354767

[hipo70125-bib-0004] Gehring, W. J. , B. Goss , M. G. Coles , D. E. Meyer , and E. Donchin . 1993. “A Neural System for Error Detection and Compensation.” Psychological Science 4, no. 6: 385–390.

[hipo70125-bib-0005] Gil‐Jardiné, C. , M. Née , E. Lagarde , et al. 2017. “The Distracted Mind on the Wheel: Overall Propensity to Mind Wandering Is Associated With Road Crash Responsibility.” PLoS One 12, no. 8: e0181327. 28771623 10.1371/journal.pone.0181327 PMC5542598

[hipo70125-bib-0006] Hargreaves, E. L. , A. T. Mattfeld , C. E. L. Stark , and W. A. Suzuki . 2012. “Conserved fMRI and LFP Signals During New Associative Learning in the Human and Macaque Monkey Medial Temporal Lobe.” Neuron 74, no. 4: 743–752. 22632731 10.1016/j.neuron.2012.03.029 PMC3969034

[hipo70125-bib-0007] Kam, J. W. Y. , E. Dao , P. Blinn , O. E. Krigolson , L. A. Boyd , and T. C. Handy . 2012. “Mind Wandering and Motor Control: Off‐Task Thinking Disrupts the Online Adjustment of Behavior.” Frontiers in Human Neuroscience 6: 329. 23248596 10.3389/fnhum.2012.00329 PMC3522104

[hipo70125-bib-0008] Ku, S. , E. L. Hargreaves , S. Wirth , and W. A. Suzuki . 2021. “The Contributions of Entorhinal Cortex and Hippocampus to Error Driven Learning.” Communications Biology 4, no. 1: 1–12. 34031534 10.1038/s42003-021-02096-z PMC8144598

[hipo70125-bib-0009] Leszczynski, M. , L. Chaieb , T. P. Reber , M. Derner , N. Axmacher , and J. Fell . 2017. “Mind Wandering Simultaneously Prolongs Reactions and Promotes Creative Incubation.” Scientific Reports 7, no. 1: 10197. 28860620 10.1038/s41598-017-10616-3 PMC5578971

[hipo70125-bib-0010] Metcalfe, J. 2017. “Learning From Errors.” Annual Review of Psychology 68: 465–489. 10.1146/annurev-psych-010416-044022 27648988

[hipo70125-bib-0011] Mormann, F. , J. Dubois , S. Kornblith , et al. 2011. “A Category‐Specific Response to Animals in the Right Human Amygdala.” Nature Neuroscience 14, no. 10: 1247–1249. 21874014 10.1038/nn.2899 PMC3505687

[hipo70125-bib-0012] Niediek, J. , J. Boström , C. E. Elger , and F. Mormann . 2016. “Reliable Analysis of Single‐Unit Recordings From the Human Brain Under Noisy Conditions: Tracking Neurons Over Hours.” PLoS One 11, no. 12: e0166598. 27930664 10.1371/journal.pone.0166598 PMC5145161

[hipo70125-bib-0013] Pourtois, G. , R. Vocat , K. N'diaye , L. Spinelli , M. Seeck , and P. Vuilleumier . 2010. “Errors Recruit Both Cognitive and Emotional Monitoring Systems: Simultaneous Intracranial Recordings in the Dorsal Anterior Cingulate Gyrus and Amygdala Combined With fMRI.” Neuropsychologia 48, no. 4: 1144–1159. 20026086 10.1016/j.neuropsychologia.2009.12.020

[hipo70125-bib-0014] Quiroga, R. Q. , Z. Nadasdy , and Y. Ben‐Shaul . 2004. “Unsupervised Spike Detection and Sorting With Wavelets and Superparamagnetic Clustering.” Neural Computation 16, no. 8: 1661–1687. 15228749 10.1162/089976604774201631

[hipo70125-bib-0015] Reber, T. P. , J. Faber , J. Niediek , J. Boström , C. E. Elger , and F. Mormann . 2017. “Single‐Neuron Correlates of Conscious Perception in the Human Medial Temporal Lobe.” Current Biology 27, no. 19: 2991–2998.e2. 28943091 10.1016/j.cub.2017.08.025

[hipo70125-bib-0016] Ridderinkhof, K. R. , M. Ullsperger , E. A. Crone , and S. Nieuwenhuis . 2004. “The Role of the Medial Frontal Cortex in Cognitive Control.” Science 306, no. 5695: 443–447. 15486290 10.1126/science.1100301

[hipo70125-bib-0017] Robertson, I. H. , T. Manly , J. Andrade , B. T. Baddeley , and J. Yiend . 1997. “Oops! Performance Correlates of Everyday Attentional Failures in Traumatic Brain Injured and Normal Subjects.” Neuropsychologia 35, no. 6: 747–758. 9204482 10.1016/s0028-3932(97)00015-8

[hipo70125-bib-0018] Schooler, J. W. , J. Smallwood , K. Christoff , T. C. Handy , E. D. Reichle , and M. A. Sayette . 2011. “Meta‐Awareness, Perceptual Decoupling and the Wandering Mind.” Trends in Cognitive Sciences 15, no. 7: 319–326. 21684189 10.1016/j.tics.2011.05.006

[hipo70125-bib-0019] Seli, P. , J. A. Cheyne , and D. Smilek . 2013. “Wandering Minds and Wavering Rhythms: Linking Mind Wandering and Behavioral Variability.” Journal of Experimental Psychology. Human Perception and Performance 39, no. 1: 1–5. 23244046 10.1037/a0030954

[hipo70125-bib-0020] Sonuga‐Barke, E. J. S. , and F. X. Castellanos . 2007. “Spontaneous Attentional Fluctuations in Impaired States and Pathological Conditions: A Neurobiological Hypothesis.” Neuroscience and Biobehavioral Reviews 31, no. 7: 977–986. 17445893 10.1016/j.neubiorev.2007.02.005

[hipo70125-bib-0021] Ullsperger, M. , C. Danielmeier , and G. Jocham . 2014. “Neurophysiology of Performance Monitoring and Adaptive Behavior.” Physiological Reviews 94, no. 1: 35–79. 24382883 10.1152/physrev.00041.2012

[hipo70125-bib-0022] Völker, M. , L. D. J. Fiederer , S. Berberich , et al. 2018. “The Dynamics of Error Processing in the Human Brain as Reflected by High‐Gamma Activity in Noninvasive and Intracranial EEG.” NeuroImage 173: 564–579. 29471099 10.1016/j.neuroimage.2018.01.059

[hipo70125-bib-0023] Wirth, S. , E. Avsar , C. C. Chiu , et al. 2009. “Trial Outcome and Associative Learning Signals in the Monkey Hippocampus.” Neuron 61, no. 6: 930–940. 19324001 10.1016/j.neuron.2009.01.012 PMC2723837

[hipo70125-bib-0024] Yaor, E. , and S. Toker . 2020. “Mind Wandering at Work: When Employees' Thoughts Drift Away.” In Stress and Quality of Working Life: Finding Meaning in Grief and Suffering, 181–194. Information Age Publishing, Inc.

[hipo70125-bib-0025] Yordanova, J. , M. Falkenstein , J. Hohnsbein , and V. Kolev . 2004. “Parallel Systems of Error Processing in the Brain.” NeuroImage 22, no. 2: 590–602. 15193587 10.1016/j.neuroimage.2004.01.040

